# Small cell prostate cancer: risks for metastatic disease

**DOI:** 10.3389/fruro.2026.1787906

**Published:** 2026-03-10

**Authors:** Essam Al-Snayyan, Jamil Qiqieh, Cameron Peres, Sharon Tan, Susan Lyons, Avery Mendelson

**Affiliations:** Internal Medicine, Henry Ford Health System, Detroit, MI, United States

**Keywords:** cancer, cell, factors, metastatic, prostate, risk, small

## Abstract

**Objective:**

To identify clinical and demographic factors associated with the presence and distribution of metastatic disease at diagnosis in patients with small cell carcinoma of the prostate (SCCP).

**Introduction:**

Small cell carcinoma of the prostate is a rare but highly aggressive subtype of prostate cancer, frequently presenting with distant metastases and poor survival outcomes. Despite its severity, population-level data examining predictors of metastatic disease at presentation remain limited. Understanding these factors may improve early detection and risk stratification.

**Methods:**

A retrospective, cross-sectional analysis was conducted using the SEER 17 registries (2000–2022). Patients with microscopically confirmed SCCP were identified using ICD-O-3 histology code 8041/3 and primary site code C61.9. Demographic, clinical, and metastatic variables were extracted. Univariate and multivariable logistic regression models were used to evaluate predictors of overall and site-specific metastases. Variables meeting a univariate threshold of p < 0.25 were included in multivariable models. Model assumptions were assessed using VIF, Tolerance, and Box–Tidwell tests.

**Results:**

A total of 541 patients were identified, of whom 71.7% presented with metastatic disease. The most common metastatic sites were bone (35.9%), liver (22.6%), lung (14.0%), and brain (3.9%). On multivariate analysis, a higher percentage of positive biopsy cores was independently associated with increased odds of metastatic disease. Younger age was associated with higher odds of brain metastasis. Lower household income and residence in metropolitan counties were associated with increased likelihood of metastatic disease, particularly bone metastases. Brain metastasis was strongly associated with concurrent liver and lung metastases, suggesting a pattern of widespread systemic involvement.

**Conclusions/discussion:**

Patients with SCCP frequently present with metastatic disease, and several clinical and socioeconomic factors influence metastatic risk. Higher tumor burden, lower income, metropolitan residence, and younger age were associated with a greater likelihood of metastasis at diagnosis. These findings highlight the importance of early diagnostic evaluation in high-risk groups and provide a foundation for improved prognostication and targeted care strategies in this rare and aggressive malignancy.

## 1 Introduction

Prostate cancer remains one of the most prevalent malignancies among men worldwide, representing a leading cause of cancer-related morbidity and mortality. While most cases are adenocarcinomas, rare histologic subtypes such as small cell carcinoma of the prostate (SCCP) represent less than 1% of diagnoses, but area associated with a markedly poorer prognosis. SCCP is characterized by its aggressive clinical course, high rate of metastases, and poor response to androgen deprivation therapy. Unlike conventional prostate adenocarcinoma, SCCP often presents disproportionately low prostate-specific antigen (PSA) levels despite extensive disease burden, reflecting its unique tumor biology and diagnostic challenges (1, 2).

Recent population-based analyses have highlighted concerning trends in the clinical outcomes of SCCP. In a large Surveillance, Epidemiology, and End Results (SEER) study, Wang et al. demonstrated that both the incidence and mortality of SCCP have increased over recent decades, with reported one-, two-, and five-year overall survival rates of 42.1%, 22.1%, and 12.5%, respectively (1). Older age, advanced disease stage, and race were identified as independent predictors of worse survival. Notably, prostate cancer exhibits the most significant racial disparity among US cancers, with black men experiencing more than a two-fold increased risk of mortality compared to all other racial groups (3). These findings show the aggressive nature of SCCP and the continued challenge of improving early detection and management. Similarly, other studies have shown that SCCP frequently presents distant metastases at diagnosis, contributing to its dismal outcomes and limited treatment options (4, 5).

Despite these insights, there remain significant gaps in understanding the epidemiology and metastatic patterns of SCCP. Specifically, few studies have systematically analyzed the relationship between clinical and demographic variables—such as age, PSA, race, marital status, and socioeconomic indicators—and the risk of metastatic disease at presentation. Furthermore, the site-specific distribution of metastases (bone, liver, lung, and brain) and their associated risk factors remain poorly characterized in population-level data. In typical prostate adenocarcinoma, the most common sites of metastasis are bone and regional lymph nodes, while SCCP is known to have a higher propensity for visceral spread to sites like the lung and liver (6). Identifying these associations is critical to improving prognostication and guiding tailored therapeutic strategies.

The present study utilizes the SEER database to comprehensively evaluate the clinicopathologic characteristics, metastatic patterns, and predictors of metastasis in patients with primary SCCP. We hypothesize that certain demographic and tumor-related variables—including age, PSA level, and tumor burden—are significantly associated with the presence and distribution of metastatic disease at diagnosis. By characterizing these factors in a large, contemporary cohort, this study aims to enhance the understanding of SCCP’s metastatic behavior and inform future risk stratification and management strategies.

## 2 Methods

### 2.1 Study design, setting, duration, and characteristics

A retrospective, population-based, cross-sectional study was conducted using data from the SEER 17 registries database (2000–2022 release). The SEER program, administered by the U.S. National Cancer Institute, encompasses approximately 34.6% of the U.S. population and provides comprehensive information on cancer incidence, patient demographics, treatment characteristics, and survival outcomes.

Patients with a confirmed diagnosis of small cell carcinoma of the prostate (SCCP) were identified using the International Classification of Diseases for Oncology, Third Edition (ICD-O-3) histology code 8041/3, and primary site code C61.9 (prostate). Only microscopically confirmed primary malignancies were included. Cases identified solely through autopsy or death certificates, and those missing data on key analytic variables related to metastatic status, were excluded.

Extracted variables included age at diagnosis, race, year of diagnosis, prostate-specific antigen (PSA) level, marital status, household income, rural–urban continuum, SEER cause-specific death classification, and presence of metastasis at diagnosis (overall and site-specific to bone, brain, liver, and lung). The primary outcome variable was metastatic status at presentation, while secondary outcomes included site-specific metastases.

### 2.2 Data processing and variable validation

Data extraction was performed using SEER*Stat software version 8.4.2 (7), and statistical analyses were conducted using IBM SPSS Statistics version 29 (IBM Corp., Armonk, NY, USA). Continuous variables were presented as mean ± standard deviation (SD) or median, while categorical variables were summarized as frequencies and percentages.

Multicollinearity among predictor variables was assessed using Variance Inflation Factor (VIF) and Tolerance statistics, with no evidence of collinearity observed (VIF < 5, Tolerance > 0.2). The Box–Tidwell transformation was applied to verify the linearity assumption of continuous predictors in relation to the logit of the outcome variable.

During univariate analysis, the Summary Stage (2000–2022) variable was excluded from the logistic regression models due to perfect collinearity with the outcome (metastatic status), which resulted in unstable parameter estimates. Additionally, PSA levels were excluded from the final multivariate analyses due to limited sample size. For the lung metastasis model, race and rural–urban continuum variables were excluded to address quasi-separation issues.

### 2.3 Statistical analysis

Descriptive analyses using percentages and means with standard deviations were performed to summarize baseline patient demographics and clinical characteristics. Predictors of metastasis were analyzed using binary logistic regression. Variables with a univariate p < 0.25 were entered into the multivariate model. Adjusted odds ratios (ORs) and 95% confidence intervals (CIs) were calculated for each covariate. Model adequacy was assessed using the Hosmer–Lemeshow goodness-of-fit test, while Cox–Snell R² and Nagelkerke R² values were used to evaluate overall model performance. For the final multivariate model predicting metastatic disease, the Cox–Snell R² was 0.138 and the Nagelkerke R² was 0.197, indicating acceptable explanatory power. All statistical tests were two-tailed, and a p-value < 0.05 was considered statistically significant.

### 2.4 Ethical considerations

The study utilized de-identified, publicly accessible data from the SEER database, which contains no personal identifiers and is freely available for research purposes. As such, this study was exempt from review by an Institutional Review Board (IRB) or ethics committee, and no informed consent was required in accordance with the U.S. Department of Health and Human Services guidelines for research using publicly available data.

## 3 Results

A total of 541 patients diagnosed with SCCP were identified from the SEER database between 2000 and 2022. The mean age at diagnosis was 70.10 ± 10.49 years, and the mean year of diagnosis was 2014 ± 6.26. The mean PSA level at diagnosis was 52.34 ng/mL, and the mean percentage of positive biopsy cores was 85.17 ± 24.37%. Among these patients, 388 (71.7%) presented with metastatic disease at diagnosis, consistent with prior reports (1). The majority of patients were White (83.2%) and married (65.8%).

The most common site of metastasis was the bone (35.9%), followed by the liver (22.6%), lung (14.0%), and brain (3.9%). Most patients resided in metropolitan areas with populations over one million (56.4%), and the majority reported a median household income between $60,000 and $99,999 (**Table 1**).

Table 1Demographics.

**Table:** 

Variable		Mean ± SD			
Age		70.10 ± 10.49			
Year of Dx		2014 ± 6.255			
PSA		52.34 ± 12.05			
Number of positive cores		9.05 ± 4.58			
Percent Positive Cores		85.17 ± 24.37			

	Frequency	Percent	Mets		P-value
Race			Non-met	Metastatic	0.501
White	450	83.2	127	323	
Black	55	10.2	13	42	
American Indian	6	1.1	3	3	
American Pacific	30	5.5	10	20	
Income					.355
<$40,000	8	1.5	4	4	
$40,000-59,999	61	11.3	16	45	
$60,000-79,999	183	33.8	59	124	
$80,000-99,999	184	34	47	137	
$100,000-119,999	73	13.5	21	152	
$120,000>	32	5.9	6	26	
Rural/Urban					0.389
Metropolitan cities with a population >1M	305	56.4	83	222	
Metropolitan cities with population 250K to 1M	126	23.3	34	92	
Metropolitan cities with population <250K	31	5.7	7	42	
Nonmetropolitan cities adjacent to metropolitan cities	45	8.3	15	30	
Nonmetropolitan cities NOT adjacent to metropolitan cities	34	6.3	14	20	
Marital status					.129
Single	74	13.7	16	58	
Married	356	65.8	108	248	
Previously Married	91	16.8	20	71	
Missing	20	3.7			
Liver mets					
No	273	50.2			
Yes	122	22.6			
Missing	146	27			
Brain mets					
No	372	68.8			
Yes	21	3.9			
Missing	148	27.4			
Lung mets					
No	311	57.5			
Yes	76	14			
Missing	154	28.5			
Bone mets					
No	202	37.3			
Yes	194	35.9			
Missing	145	26.8			
Any mets					
No	153	30.3			
Yes	352	69.7			
Multiple mets					
No	153	28.3			
Yes	388	71.7			

### 3.1 Univariate analysis

In the univariate logistic regression analysis (**Table 2**), several variables demonstrated statistically significant associations with the presence of metastases at diagnosis. Older age was associated with reduced odds of both bone metastases (p = 0.021) and brain metastases (p = 0.042). An earlier year of diagnosis was significantly associated with increased odds of overall metastatic disease (p = 0.002). Higher PSA levels were associated with greater odds of bone (p = 0.004) and liver metastases (p = 0.004). A higher percentage of biopsy cores positive for malignancy was also associated with increased odds of overall metastasis (p = 0.049).

**Table 2:** Overall metastasis logistic regression, univariate analysis.

Univariate analysis					
Variable	Overall mets	Bone	Brain	Liver	Lung
Age (years)	0.984 (0.966-1.002) p=0.077	0.977 (0.958-0.997)* **p=0.021**	0.956 (0.916-0.998)* **p=0.042**	0.997 (0.976-1.018) p=0.793	0.998 (0.974-1.024) p=0.906
Year of diagnosis	1.047 (1.017-1.079)* **p=0.002**	1.031 (0.976-1.088) p=0.274	1.111 (0.974-1.266) p=0.117	1.044 (0.984-1.108) p=0.156	1.003 (0.936-1.075) p=0.932
PSA (2018+)	1.021 (0.999-1.044) p=0.057	1.022 (1.007-1.038)* **p=0.004**	0.999 (0.993-1.005) p=0.781	1.002 (0.999-1.004) p=0.133	1.001 (0.999-1.003) p=0.444
Percentage of biopsy cores positive for malignancy	1.015 (1.00-1.03)* **p=0.049**	1.013 (0.997-1.029) p=0.109	1.002 (0.967-1.038) p=0.913	1.018 (0.996-1.039) p=0.106	1.032 (0.993-1.072) p=0.107
Race (ref: White)					
Black	1.270 (0.66-2.446) p=0.474	1.343 (0.726-2.484) p=0.348	N/A	N/A	0.465 (0.117-1.225) p=0.121
American Indian/Alaskan Native	1.393 (0.0780-1.974) p=0.257	0.723 (0.119-4.385) p=0.724	N/A	N/A	0.954 (0.105-8.680) p=0.967
Asian/Pacific Islander	0.786 (0.3580-1.726) p=0.549	1.183 (0.507-2.759) p=0.697	N/A	N/A	1.060 (0.379-2.962) p=0.912
Marital status (ref: Single)					
Married	0.633 (0.348-1.152) p=0.134	0.482 (0.269-0.862)* **p=0.014**	1.481 (0.379-5.794) p=0.572	0.933 (0.508-1.715) p=0.823	0.987 (0.487-2.000) p=0.972
Previously married	0.996 (0.470-2.109) p=0.956	0.779 (0.381-1.594) p=0.494	0.727 (0.224-2.360) p=0.596	0.917 (0.432-1.945) p=0.822	0.753 (0.304-1.864) p=0.540
Median household income (ref: < $40k)					
$40–59k	2.813 (0.628- 12.589) p=0.176	2.963 (0.317-27.674) p=0.085	1.050 (0.095-11.558) p=0.968	0.688 (0.110-4.288) p=0.688	0.385 (0.033-4.445) p=0.444
$60–79k	2.102 (0.508- 8.696) p=0.305	4.632 (0.527-40.743) p=0.167	0.404 (0.083-4.977) p=0.263	0.758 (0.133-4.319) p=0.755	0.818 (0.090-7.414) p=0.858
$80–99k	2.917 (0.701-12.119) p=0.278	5.615 (0.639-49.324) p=0.120	0.252 (0.066-0.969)* **p=0.045**	0.894 (0.157-5.071) p=0.899	1.584 (0.178-14.064) p=0.680
$100–119k	2.476 (0.506-10.831) p=0.228	4.815 (0.526-44.046) p=0.164	0.159 (0.037-0.685)* **p=0.014**	1.176 (0.197-7.011) p=0.858	1.463 (0.156-13.765) p=0.739
≥$120k	4.333 (0.836-22.469) p=0.081	7.500 (0.759-74.157) p=0.085	0.315 (0.065-1.531) p=0.152	1.571 (0.242-10.217) p=0.636	3.333 (0.337-32.959) p=0.303
Rural/Urban (ref: Metropolitan cities with ≥1M population)					
Metropolitan cities with Population 250k–1M	1.012 (0.634-1.64) p=0.058	0.877 (0.546-1.408) p=0.585	0.534 (0.110-2.594) p=0.437	0.934 (0.563-1.548) p=0.791	0.925 (0.519-1.652) p=0.793
Metropolitan cities with Population <250k	1.282 (0.532-3.087) p=0.395	1.078 (0.463-2.512) p=0.862	0.458 (0.079-2.663) p=0.385	0.881 (0.347-2.238) p=0.790	0.764 (0.247-2.365) p=0.640
Nonmetropolitan cities adjacent to metropolitan cities	0.748 (0.383-1.460) p=0.395	0.528 (0.240-1.162) p=0.113	1.048 (0.135-8.131) p=0.965	0.881 (0.347-2.238) p=0.282	0.246 (0.056-1.068) p=0.061
Nonmetropolitan cities NOT adjacent to metropolitan cities	0.534 (0.258-1.106) p=0.91	0.376 (0.150-0.942)* **p=0.037**	1.222 (0.187-7.975) p=0.834	0.403 (0.133-1.222) p=0.108	0.156 (0.021-1.189) p=0.073
Bone Mets			0.866 (0.359-2.088) p=0.749	1.926 (1.246-2.979)* **p=0.003**	1.695 (1.019-2.820)* **p=0.045**
Brain Mets		1.155 (0.479-2.785) p=0.749		2.634 (1.087-6.382)* **p=0.032**	0.372 (0.148-0.933)* **p=0.035**
Liver Mets		1.926 (1.246-2.979)* **p=0.003**	0.380 (0.157-0.920)* **p=0.032**		3.386 (2.005-5.719)* **p= <0.001**
Lung Mets		1.695 (1.019-2.820)* **p=0.042**	0.372 (0.148-0.933)* **p=0.035**	3.386 (2.005-5.119)* **p= <0.001**	

Cells labeled N/A were excluded due to limited sample size.

Cells shaded grey were excluded due to logical redundancy.

Bolded values indicate that the results were statistically significant.Symbol * also indicates the result is statistically significant.

Marital status showed a significant association, with married individuals demonstrating lower odds of bone metastasis compared with single individuals (p = 0.014). Among income categories, patients with higher household income were less likely to have brain metastases, specifically those earning $80,000–99,999 (p = 0.045) and $100,000–119,999 (p = 0.014), compared with those earning less than $40,000. Lastly, residing in nonmetropolitan nonadjacent areas was associated with lower odds of both overall (p = 0.037) and bone metastases (p = 0.037).

### 3.2 Multivariate analysis

In the multivariate logistic regression analysis (**Table 3**), after controlling for relevant covariates, several variables remained significantly associated with metastatic disease across specific sites. A higher percentage of biopsy cores positive for malignancy was independently associated with increased odds of overall metastasis (p = 0.028). Older age was significantly associated with lower odds of brain metastasis (p = 0.035). Socioeconomic status was significantly associated with metastatic patterns. Patients with a median household income between $40,000 and $59,999 had higher odds of presenting with bone metastases (p = 0.021), suggesting that lower–middle income status may be linked to increased metastatic risk. Conversely, patients in the highest income bracket ($100,000–$119,999) showed lower odds of brain metastases (p = 0.003), suggesting an inverse relationship between socioeconomic status and brain involvement. Similarly, residence in nonmetropolitan areas was associated with a decreased likelihood of bone metastasis; both nonmetro adjacent (p = 0.038) and nonmetro nonadjacent (p = 0.043) locations showed reduced odds compared to metropolitan regions. Additionally, brain metastasis showed a potential association with increased odds of liver metastasis (p = 0.035) and lung metastasis (p = 0.041), though the wide confidence intervals suggest this relationship requires further validation in larger cohorts.

**Table 3:** Overall metastasis logistic regression.

Multivariate analysis					
Variable	Overall mets	Bone	Brain	Liver	Lung
Age (years)	0.981 (0.936-1.028) p=0.416	0.967 (0.926-1.009) p=0.123	0.949 (0.904-0.996)* **p=0.035**	REF	N/A
Year of diagnosis	1.022 (0.888-1.176) p=0.762	REF	1.098 (0.951-1.268) p=0.203	1.061 (0.929-1.213) p=0.382	N/A
Percentage of biopsy cores positive for malignancy	1.020 (1.002-1.039)* **p=0.028**	1.017 (0.998-1.035) p=0.080	REF	1.014 (0.992-1.036) p=0.202	N/A
Marital status (ref: Single)	REF	REF	REF	REF	N/A
Married	0.239 (0.045-1.255) p=0.091	0.352 (0.102-1.213) p=0.098	REF	N/A	N/A
Previously married	0.641 (0.084-4.897) p=0.668	0.789 (0.153-4.064) p=0.776	REF	N/A	N/A
Median household income (ref: < $40k)	REF	REF	REF	REF	
$40–59k	1.042 (0.049-21.950) p=0.979	**1.234** (0.070-21.6)* **p=0.021**	0.448 (0.036-5.665) p=0.535	REF	N/A
$60–79k	0.845 (0.038-18.554) p=0915	1.881 (0.101-35.071) p=0.179	0.211 (0.020-2.263) p=0.199	REF	N/A
$80–99k	0.871 (0.034-22.211) p=0.934	1.813 (0.088-37.126) p=0.149	0.132 (0.011-1.523) p=0.105	REF	N/A
$100–119k	0.743 (0.026-21.041) p=0.862	0.913 (0.041-20.547)* **p=0.003**	0.245 (0.019-3.120) p=0.279	REF	N/A
≥$120k	0.649 (0.015-27.511) p=0.821	2.021 (0.067-60.955) p=0.164	0.543 (0.042-6.986) p=0.639	REF	N/A
Rural/Urban (ref: ≥1M population)					
Population 250k–1M	0.915 (0.289-2.904) p=0.881	0.943 (0.325-2.741) p=0.012	N/A	0.564 (0.186-1.708) p=0.311	Limited Sample Size
Population <250k	0.838 (0.121-5.783) p=0.857	1.584 (0.248-10.107) p=0.237	N/A	1.007 (0.173-5.875) p=0.994	Limited Sample Size
Nonmetropolitan cities adjacent to metropolitan cities	0.672 (0.100-4.513) p=0.683	0.836 (0.138-5.066)* **p=0.038**	N/A	0.733 (0.151-3.548) p=0.699	Limited Sample Size
Nonmetropolitan cities NOT adjacent to metropolitan cities	0.707 (0.106-4.703) p=0.720	0.820 (0.127-5.287)* **p=0.043**	N/A	0.389 (0.058-2.587) p=0.329	Limited Sample Size
Bone Mets			N/A	1.876 (0.770-4.571) p=0.166	1.392 (0.445-4.358) p=0.570
Brain Mets		N/A		10.136 (1.174-87.534)* **p=0.035**	9.225 (1.100-77.370)* **p=0.041**
Liver Mets		2.003 (0.787-5.101) p=0.145	2.104 (0.819-5.409) p=0.123		1.962 (0.612-6.287) p=0.257
Lung Mets		1.857 (0.553-6.235) p=0.317	2.483 (0.886-6.957) 0.084	1.966 (0.600-6.439) p=0.264	

REF: Reference category for categorical variables. Each column represents an independent multivariable regression model evaluating predictors of the specified metastatic outcome (overall metastasis, bone, brain, liver, or lung metastases). Because these are separate models, reference categories are defined within each analysis. “REF” denotes the baseline comparator for that specific model.

Variables controlled for in the overall metastasis model:

Age, year of diagnosis, marital status, median household income, rural–urban residence, and percentage of positive core biopsies.

Variables controlled for in the bone metastasis model:

Age, marital status, median household income, rural–urban residence, liver metastases, lung metastases, and percentage of positive core biopsies.

Variables controlled for in the brain metastasis model:

Age, year of diagnosis, median household income, liver metastases, and lung metastases.

Variables controlled for in the liver metastasis model:

Year of diagnosis, rural–urban residence, bone metastases, brain metastases, lung metastases, and percentage of positive core biopsies.

Variables controlled for in the lung metastasis model:

Rural–urban residence, bone metastases, brain metastases, liver metastases, and percentage of positive core biopsies.

Metastatic site variables were mutually adjusted for the presence of other metastatic sites, except when the site served as the outcome variable, in which case it was not included as a covariate.

Covariates were retained based on model stability and statistical contribution within each outcome-specific regression. Brain metastasis models required variable reduction due to lower event counts.

Cells marked “N/A” indicate variables not included in the model due to model structure, limited sample size, or model instability. Cells shaded grey were excluded due to logical redundancy (variable identical to the model outcome). PSA and race variables were evaluated but not included in final multivariable models due to incomplete data and lack of contribution to model stability.

Bolded values indicate that the results were statistically significant.Symbol * also indicates the result is statistically significant.

## 4 Discussion

Analysis of data from the SEER database from 2000 to 2022 revealed that 71.7% of patients with SCCP presented with metastatic disease at the time of diagnosis. There were several variables that included both clinical and demographic factors that were associated with an increased likelihood of presenting with metastatic disease. These included a higher percentage of positive core biopsies, lower household income, residence in metropolitan counties, and metastatic disease to the brain associated with younger patient age. These findings are consistent with SCCP which has been seen to metastasize earlier with its neuroendocrine traits and has a poor response to androgen deprivation therapy (1, 2, 4, 5). This propensity for early metastatic spread corresponds with the tumor’s underlying biology: SCCP commonly shows absent PSA and PSAP staining, a hallmark of its dedifferentiated neuroendocrine phenotype and a driver of its highly aggressive clinical course (8).

Previous studies have shown that patients with SCCP who present with metastatic disease have worse outcomes than those with localized disease. Wang et al. reported one- and two-year survival rates of 42.1% and 22.1% for patients with SCCP that presented with metastatic disease at presentation, thus being one of the strongest predictors of early mortality (1). Other studies have shown five-year survival rates below 15% for patients diagnosed with advanced stage disease at presentation (4, 5). These investigations have also shown the importance of understanding which patients are at higher probability for presenting with metastatic disease, as early recognition remains one of the few modifiable points in the disease course.

Literature examining the clinical and socioeconomic predictors of metastatic presentation in SCCP remains sparse, particularly regarding large-scale cohorts. While Weiner et al. utilized the National Cancer Database to identify SES factors linked to metastatic prostate cancer broadly, their analysis focused on adenocarcinoma and not SCCP (9). Their findings showed that Lower SES, race/ethnicity, and having Medicaid or no insurance were each independently associated with higher odds of presenting with metastases at the time of prostate cancer diagnosis. In contrast, our study evaluates metastatic patterns across multiple organ sites to provide a more granular perspective and integrates these sociodemographic variables within our models.

This study builds on earlier work by evaluating a broader based U.S. population with a wider range of sociodemographic measures and analyzing metastatic patterns across multiple organ sites. The results of this study show that the percentage of biopsy cores positive for prostate cancer is an indicator of intraprostatic tumor burden and was independently associated with the presence of metastatic disease. These results align with the aggressive growth pattern of typical SCCP which frequently involves diffuse infiltration of the prostate and early hematogenous spread even with relatively low PSA levels (2). The association between greater tumor burden and distant metastasis at diagnosis reinforces the importance of rapid diagnostic workup and staging once SCCP is suspected.

In this study, younger age was found to be independently associated with metastatic brain disease at diagnosis. There is significant uncertainty regarding why younger individuals with SCCP may present with a higher prevalence of brain mets. Potentially this may be due to the biologically aggressive neuroendocrine variants being overrepresented in younger patients, or that a more robust vascular or proliferative tumor phenotype contributes to earlier central nervous system metastasis. Similar age-related patterns have been observed in other high-grade neuroendocrine cancers, though confirmatory molecular studies are needed to better define this relationship. Given the small sample size of patients with brain metastases in this study, these findings should be interpreted as suggestive but require further validation. Genomic profiling of small cell lung cancer, a tumor with similar morphology, consistently shows the bi-allelic inactivation of the TP53 and RB1 tumor suppressor genes, which are also frequently observed in prostatic SCC (10). Future genomic profiling stratified by age, utilizing larger multi-institutional datasets, will be necessary to determine whether these alterations occur more frequently in younger patients.

We also found that lower household income and residence in metropolitan areas were associated with a greater probability of metastatic disease at presentation. This was particularly associated with a higher incidence of metastatic bone disease. The results showed that married men were less likely to present with metastatic bone disease. This highlights a vulnerable demographic within the SCCP population—single men—who may lack the domestic ‘safety net’ that often leads to earlier symptomatic evaluation. Unlike age or tumor burden, which are characteristics specific to the patient and cancer, income level and geographic region reflect the broader environment where the patient lives and receives care. Lower socioeconomic status may reflect a reduction in access to healthcare resources, delayed diagnostic evaluation, or differences in an individual’s ability to seek medical care. County level income metrics are not perfect as it is hard to capture an individual’s full socioeconomic circumstance, but it does provide meaningful information on community level disparities. Socioeconomic disparities in prostate cancer outcomes are often tied to differences in access to health and screening services, leading to a later stage of diagnosis (11). The racial disparity in prostate cancer, which is particularly severe in younger Black men, has been partially linked to shorter CAG repeats in the androgen receptor gene, a factor associated with more aggressive disease (3). As seen in studies of other cancers, socioeconomic disadvantages have repeatedly been associated with more advanced disease at presentation. These results suggest that SCCP may follow a similar pattern, and that targeted outreach in underserved populations may help facilitate an earlier diagnosis.

The study also observed an association between metastatic brain disease and the presence of liver and lung metastases, though the wide confidence intervals for these site-specific estimates suggest this relationship should be interpreted with caution. This may suggest that patients with brain metastasis are more likely to have widespread systemic disease which is consistent with previous descriptions of SCCP and a high likelihood of multiorgan metastasis (4, 5). Whether this reflects true biological clustering or simply a marker of disease severity remains unclear, but it shows the importance of thorough staging, particularly in symptomatic individuals or those with radiologic evidence of metastatic disease.

Although the present investigation was not designed to guide clinical management, the identification of high-risk subgroups may have practical implications. For example, patients with extensive core positivity or lower socioeconomic status may benefit from expedited staging, early referral to multidisciplinary oncology teams, or enhanced counseling regarding the likelihood of discovering metastatic disease. Whether these patients would benefit from additional imaging, earlier systemic therapy initiation, or alternate diagnostic pathways is not addressed by this study but represents an important area for future research.

## 5 Limitations

This study has several limitations. As with all analyses using SEER data, we were unable to verify pathologic diagnoses or confirm the accuracy of metastatic site reporting. Key variables such as Ki-67 index, tumor size, symptom duration, or genomic profiles were not available, limiting our ability to assess biological contributors to metastatic behavior. Additionally, in the multivariate analysis, PSA and race had to be excluded due to This study’s socioeconomic status was completed using county median household income which may not fully capture the individual’s access to care, insurance status, or delays to diagnostic evaluations. Additionally, county data may not capture intracounty variation. Income differences in metastatic presentation may be due to community access to health care versus a patient with specific socioeconomic status.

The analysis focused exclusively on metastatic status at presentation; treatment patterns and long-term oncologic outcomes were not evaluated and should be explored in subsequent studies. Finally, despite utilizing a large population-based cohort, the rarity of SCCP resulted in limited power for certain site-specific analyses. This is reflected in the wide confidence intervals observed in some multivariate models (e.g., brain metastasis), which should be interpreted cautiously.

Another limitation noted in our study relates to the extent of missing data for site-specific metastases, which approached 25–30% for several metastatic sites. This is largely attributable to the SEER program’s longitudinal data collection standards, as these specific variables were not consistently recorded until 2010, whereas this study’s cohort commences in 2000 (12). Furthermore, information is more likely to be incomplete in earlier diagnostic years such as in patients without comprehensive staging imaging, or patients with clinically aggressive disease where full site documentation may not have been performed. Patients with more advanced or symptomatic diseases would more likely have complete staging which can bias estimates toward stronger associations between clinical or sociodemographic factors and metastatic involvement. Consequently, our findings may overestimate the strength of association between certain risk factors and metastatic involvement, as the cohort with complete data is likely skewed toward more symptomatic and advanced presentations.

While PSA is a key clinical marker, it was excluded from the final multivariable models due to a significant proportion of missing values in the SEER database. This would have compromised the model’s stability and power. Given that SCCP often presents with low PSA levels despite advanced disease, the percentage of positive biopsy cores was utilized as a primary measure of tumor burden. However, the absence of PSA in the adjusted analysis limits the accountability for unmeasured confounding related to PSA. The observed findings should then be interpreted as associations that do not account for potential variations in baseline PSA levels.

## 6 Conclusion

In summary, higher tumor burden, lower socioeconomic status, metropolitan residence, non-married status, and younger age were all associated with an increased incidence of metastatic disease at the time of presentation of SCCP. As a retrospective, cross-sectional study evaluating metastatic disease status at the time of diagnosis, this analysis cannot assess disease progression over time nor establish causal relationships between identified factors and metastatic presentations. While management recommendations are beyond the scope of this analysis, these findings identify specific patient subgroups that may warrant heightened clinical suspicion and expedited staging or referral to improve early detection. These findings present further data demonstrating both clinical and demographic factors linked to patients who present with metastatic SCCP, highlighting opportunities for improved detection and risk stratification in this disease.

## Data Availability

The original contributions presented in the study are included in the article/**Supplementary Material**. Further inquiries can be directed to the corresponding author.
